# Correction: Collective forces of tumor spheroids in three-dimensional biopolymer networks

**DOI:** 10.7554/eLife.59538

**Published:** 2020-06-02

**Authors:** Christoph Mark, Thomas J Grundy, Pamela L Strissel, David Böhringer, Nadine Grummel, Richard Gerum, Julian Steinwachs, Carolin C Hack, Matthias W Beckmann, Markus Eckstein, Reiner Strick, Geraldine M O'Neill, Ben Fabry

Mark C, Grundy TJ, Strissel PL, Böhringer D, Grummel N, Gerum R, Steinwachs J, Hack CC, Beckmann MW, Eckstein M, Strick R, O'Neill GM, Fabry B. 2020. Collective forces of tumor spheroids in three-dimensional biopolymer networks. *eLife* **9**:e51912. doi: 10.7554/eLife.51912.

Published 30, April 2020

We have identified errors in the Material and Methods section of the manuscript – under the heading “Primary breast cancer spheroid and tumoroid embedding”. These errors were unintentional and have been rectified. See below.

Original text:

- “FluoSphere polystyrene beads (1 µm diameter, Thermo Fisher Scientific) are carefully suspended, without forming bubbles, in 1.2 mg/ml collagen solution at a concentration of 2x10^8^ beads/ml. 650 µl of this mixture is poured into one well of a 6-well plate […]
- Once the spheroids/tumoroids have settled to the base of the tube, excess media is aspirated away and spheroids/tumoroids are resuspended in 650 µl of the 1.2 mg/ml collagen/bead mixture. […]”

Corrected text:

- “**Silica beads (4 µm diameter, Kisker Biotech)** are carefully suspended, without forming bubbles, in 1.2 mg/ml collagen solution at a concentration of 2x10^8^ beads/ml. **1300 µl** of this mixture is poured into one well of a 6-well plate […]
- Once the spheroids/tumoroids have settled to the base of the tube, excess media is aspirated away and spheroids/tumoroids are resuspended in **1300 µl** of the 1.2 mg/ml collagen/bead mixture. […]”

The article has been corrected accordingly.

